# ReIPS: A Secure Cloud-Based Reputation Evaluation System for IoT-Enabled Pumped Storage Power Stations

**DOI:** 10.3390/s23125620

**Published:** 2023-06-15

**Authors:** Yue Zong, Yuechao Wu, Yuanlin Luo, Han Xu, Wenjian Hu, Yao Yu

**Affiliations:** 1 Power China Huadong Engineering Corporation Limited, Hangzhou 311122, China; zong_y2@hdec.com (Y.Z.); wu_yc@hdec.com (Y.W.); luo_yl2@hdec.com (Y.L.); xu_h@hdec.com (H.X.); 2 School of Computer Science and Engineering, Northeastern University, Shenyang 110819, China; neuhwj@163.com; 3 Key Laboratory of Intelligent Computing in Medical Image, Ministry of Education, Northeastern University, Shenyang 110819, China

**Keywords:** pumped storage power stations (PSPSs), intelligent inspection devices, reputation evaluation, cloud platform, internal attack detection

## Abstract

Reputation evaluation is an effective measure for maintaining secure Internet of Things (IoT) ecosystems, but there are still several challenges when applied in IoT-enabled pumped storage power stations (PSPSs), such as the limited resources of intelligent inspection devices and the threat of single-point and collusion attacks. To address these challenges, in this paper we present *ReIPS*, a secure cloud-based reputation evaluation system designed to manage intelligent inspection devices’ reputations in IoT-enabled PSPSs. Our *ReIPS* incorporates a resource-rich cloud platform to collect various reputation evaluation indexes and perform complex evaluation operations. To resist single-point attacks, we present a novel reputation evaluation model that combines backpropagation neural networks (BPNNs) with a point reputation-weighted directed network model (PR-WDNM). The BPNNs objectively evaluate device point reputations, which are further integrated into PR-WDNM to detect malicious devices and obtain corrective global reputations. To resist collusion attacks, we introduce a knowledge graph-based collusion device identification method that calculates behavioral and semantic similarities to accurately identify collusion devices. Simulation results show that our *ReIPS* outperforms existing systems regarding reputation evaluation performance, particularly in single-point and collusion attack scenarios.

## 1. Introduction

Pumped storage power stations (PSPSs) are essential for energy storage and grid stability [1]. These facilities efficiently store excess electricity during low-demand periods and release it during high-demand periods [2,3]. Safety is a top priority in PSPSs for prevention of accidents, equipment failures, and environmental risks. This necessitates robust safety measures, reliable control systems, and diligent monitoring to ensure the smooth operation and protection of both personnel and the surrounding ecosystem [4]. The progress in Internet of Things (IoT) technology has significantly impacted PSPSs, where intelligent inspection devices can seamlessly connect to offer managers valuable services, such as efficient data collection and quick anomaly detection [5,6,7]. However, due to its highly decentralized, open, and dynamic characteristics, the IoT network is vulnerable to attacks from both external and internal sources, especially in PSPSs [8]. Although authentication, firewall, and cryptography technologies effectively defend against external attacks, they are ineffective against internal attacks launched by verified but misbehaving devices within the IoT network [9,10,11]. Therefore, it is essential to design a reliable solution that can detect internal attacks and isolate malicious devices to ensure the security of IoT-enabled PSPSs.

To provide secure and high-quality inspection services, intelligent inspection devices can rely on trust relationships for information sharing, thus avoiding interaction with malicious devices [12,13]. Reputation evaluation mechanisms enable each device to evaluate another device’s point reputation based on their interactions [14]. The point reputation serves as a reference for other devices to determine whether the target device is trustworthy for future interactions. Moreover, each device’s point reputations evaluated by multiple peers can be further aggregated into a global reputation for malicious device detection [15]. Therefore, reputation evaluation mechanisms can promote healthy interactions between devices and identify malicious ones.

However, there are still several unique challenges to be addressed when applying reputation evaluation in IoT-enabled PSPSs: (1) the limited computational and storage resources of intelligent inspection devices hinder the collection of adequate reputation evaluation indexes, hampering accurate and objective reputation calculations. (2) IoT-enabled PSPSs are susceptible to single-point attacks, where a single malicious device assigns false point reputations to well-behaved devices, thereby misleading others in their trustworthy judgments. (3) Collusion attacks pose a significant threat in IoT-enabled PSPSs, as multiple malicious devices can collaborate to invalidate malicious device detection. This can be achieved by slandering well-behaved devices‘ reputations or exaggerating partners’ reputations.

Our contributions. In this paper, we propose *ReIPS*, a secure cloud-based reputation evaluation system, to address the above-mentioned challenges of resource limitations and detection of malicious devices that launch single-point or collusion attacks. Our research aims to enhance the security and accuracy of reputation evaluation in IoT-enabled PSPSs. In particular, a cloud platform is introduced to enable complex reputation evaluation operations and behavioral analysis in IoT-enabled PSPSs, including point reputation evaluation, global reputation calculation, and collusion device identification. The main contributions of this paper are as follows.

- To improve the accuracy and objectivity of point reputation evaluation, we propose a multidimensional evaluation index system and a point reputation evaluation model based on backpropagation neural networks (BPNNs) that establishes nonlinear mappings from the indexes to corresponding point reputations.
- We introduce the point reputation-weighted directed network model (PR-WDNM) to visualize the reputation evaluation relationships between devices. Based on PR-WDNM, we propose a new weighted averaging method for point reputations, where device credibility is used as an adaptive weight to obtain the corrective global reputation. Additionally, device credibility serves as a metric for effectively detecting malicious devices that launch single-point attacks.
- To accurately identify malicious devices involved in collusion attacks, we propose a knowledge graph-based collusion device identification method. Based on the constructed knowledge graph, we can calculate and fuse behavioral and semantic similarities to identify collusion devices with the same attributes and malicious behaviors.
- Extensive simulation results demonstrate that our *ReIPS* outperforms existing benchmarks in terms of reputation evaluation performance under both single-point and collusion attack scenarios.

Organization. The remainder of this paper is organized as follows. Section 2 describes the status of the relevant research. Section 3 illustrates the system model of our *ReIPS* and the threat model. Section 4 and Section 5 present the details of our proposed reputation evaluation method and collusion device identification method, respectively. Simulation results and discussions are shown in Section 6. Section 7 concludes this paper and provides suggestions for future research directions.

## 2. Related Work

Reputation evaluation is an effective security measure for protecting IoT networks against internal attacks. Unlike traditional security measures that focus solely on external attacks, reputations enable each IoT device to identify trustworthy devices for interaction and service acquisition, thereby reducing the risk of attacks within IoT networks [16,17,18].

Point reputation refers to an IoT device’s reputation evaluated by another device based on their interactions. IoT devices can be considered as nodes in IoT networks. Zhao et al. [19] proposed an exponential-based reputation evaluation system that considers the number of interactions as the evaluation index. Rongfei et al. [20] calculated the probability of a node successfully interacting with others and used it as the index to measure the node’s reputation. However, these approaches only consider a single reputation evaluation index, making it difficult to comprehensively and accurately reflect the actual reputation status of nodes in the network.

To strengthen the credibility and applicability of reputations, recent studies have proposed global reputation evaluation approaches that evaluate the global reputation of each IoT device by aggregating its point reputations from multiple peers. Basu et al. [21] defined the global reputation of a transponder as the average of the reputations given by other nodes in the network. Wang et al. [22] used global reputation to measure a user’s trustworthiness, defined as the average of all feedback obtained from the user’s interactions. Okba et al. [23] proposed that a service provider’s global reputation depends on the evaluations provided by all clients for service quality. However, these approaches ignore that posting false point reputations may positively or negatively bias other normal nodes’ global reputations, making it difficult to detect individual malicious nodes or collusive groups in the network. To resist collusion attacks on reputation evaluation, Liu et al. [24] proposed an unfair rater detection approach based on rating behavior similarity. However, the approach failed to address the issue of sparse rating data in actual networks, leading to low accuracy in calculating behavioral similarity and an inability to discover covert collusion nodes. Therefore, it is crucial for IoT-enabled PSPSs to establish a secure and reliable reputation evaluation process that yields accurate and objective results.

Moreover, we note that applying emerging technologies such as cloud computing and knowledge graphs to reputation evaluation systems has significant potential to improve efficiency and accuracy. The authors in [25,26] have emphasized the integration of external resources, such as cloud servers and edge infrastructures, to support a wide range of IoT services. This integration provides a practical solution for conducting complex reputation evaluation operations and behavioral analysis in resource-constrained PSPSs. A knowledge graph can map out the reputation evaluation relationships between devices in IoT-enabled PSPSs, assisting in detecting collusion attacks targeted at the reputation evaluation system. Mature software, databases, and algorithms are already available to support ontology construction, graph storage, and knowledge vectorization in knowledge graphs, such as Protégé, Neo4j, and Translating Embeddings (TransE). Protégé provides convenient tools such as class, relationship, and property models for users, who can create and modify ontologies using a visual interface [27]. Neo4j is a graph database suitable for mapping entities and relationships, handling highly connected data, and providing excellent query and storage performance [28]. TransE is a typical knowledge graph embedding algorithm that maps entities and relationships to a low-dimensional vector space, enabling the computation of the behavioral similarity between entities [29]. Therefore, we will design a secure reputation evaluation system for IoT-enabled PSPSs, considering the support of these emerging technologies regarding resources and efficiency.

## 3. System Model

In this section, we provide details of the system framework of our *ReIPS* and the threat model considered in this paper.

### 3.1. System Framework

**Figure 1:**
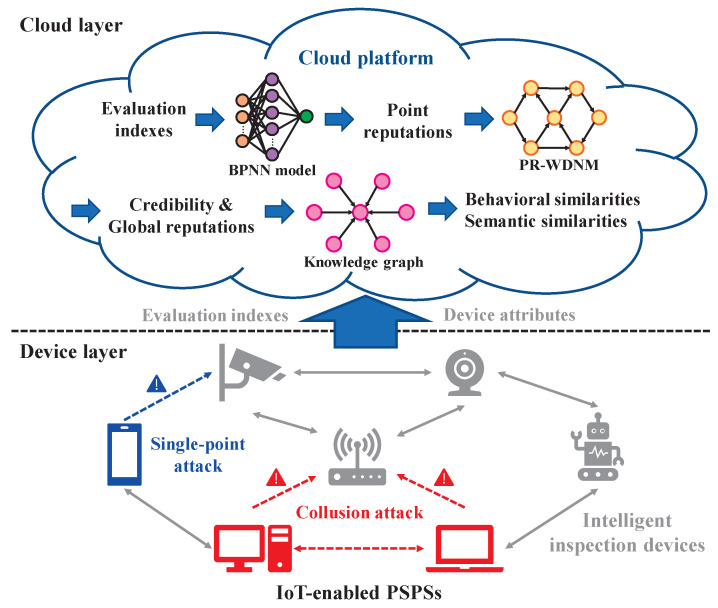
System framework of our *ReIPS*.

The system framework of our *ReIPS* is illustrated in Figure 1, designed to enable efficient reputation evaluation and behavioral analysis for intelligent inspection devices in IoT-enabled PSPSs. The framework comprises two layers: the device layer and the cloud layer.

(1) Device Layer: This layer comprises various intelligent inspection devices, such as surveillance cameras, wheeled robots, and laptops. They work collaboratively to perform functions such as comprehensive information collection, status analysis, and anomaly recognition of energy facilities in PSPSs. The devices upload multidimensional evaluation indexes to the cloud platform for reputation evaluation.

(2) Cloud Layer: The cloud layer comprises a centralized cloud server that provides sufficient computing and storage resources for reputation evaluation and behavioral analysis in IoT-enabled PSPSs. It utilizes a BPNN model to obtain point reputations of mutual evaluations between devices. These point reputations are then aggregated using our PR-WDNM to obtain each device’s credibility and global reputation, assisting the administrator in detecting single-point attacks within IoT-enabled PSPSs. Furthermore, the cloud platform constructs a knowledge graph by incorporating device attributes, interaction relationships, and reputations. This knowledge graph can assist the administrator in calculating behavioral and semantic similarities between devices for collusion attack detection.

### 3.2. Threat Model

In this paper, we consider two typical types of attacks on the reputation evaluation system in IoT-enabled PSPSs: single-point attacks and collusion attacks. Their descriptions are as follows.

- Single-Point Attacks: This type of attack involves a single malicious device providing false point reputations to other well-behaved devices to influence their trustworthiness in future interactions and global reputations.
- Collusion Attacks: In this type of attack, multiple malicious devices collaborate to slander the point reputations of well-behaved devices or exaggerate the point reputations of their partners. The goal of collusion attacks is to disrupt the detection of malicious devices and influence global reputations.

## 4. Reputation Evaluation Model

This section presents our proposed reputation evaluation model for point and global reputation based on BPNNs and PR-WDNM. Our model employs nonlinear mapping and adaptive weighted aggregation to improve the reliability and accuracy of reputation evaluation while effectively detecting malicious devices that launch single-point attacks.

### 4.1. Evaluation Index System

In our *ReIPS*, we present a multidimensional reputation evaluation index system to ensure comprehensive, objective, and accurate reputation evaluations for each device. This system includes two categories of evaluation indexes: inherent attribute indexes and performance attribute indexes. The inherent attribute indexes capture the relevance of the services provided by the device, encompassing the specification, agreement, and description of the services. On the other hand, the performance attribute indexes assess the characteristics exhibited by the device during interactions, such as response time, throughput, latency, link success rate, availability, and reliability. To provide a formal understanding of these reputation evaluation indexes, we present their specific definitions in Table 1. These indexes will be used as input terms to our designed BPNN model to obtain target point reputation through nonlinear mappings.

### 4.2. Point Reputation Evaluation Method

We propose a point reputation evaluation method based on BPNNs for intelligent inspection devices deployed in IoT-enabled PSPSs. The point reputation evaluation is a complex and dynamic process that depends on multiple indexes, making it challenging to represent the mapping relationship between these indexes and the corresponding point reputation using a specific mathematical function. Therefore, our proposed method uses the BPNN model to establish clear and nonlinear mappings from multidimensional evaluation indexes to the point reputation.

The point reputation evaluation process involves the following steps. First, the reputation evaluation indexes are collected from the devices by the cloud platform. These indexes and prior knowledge are then divided into training and test sets. Second, a BPNN model is designed on the cloud platform and trained using the training set. Third, the BPNN model is tested using the test set to analyze the accuracy of the point reputation evaluation, ensuring that it meets the usage requirements for IoT-enabled PSPSs. Finally, the trained BPNN model is deployed in the cloud platform to comprehensively and objectively evaluate each device’s point reputation. The model takes our defined evaluation indexes as input and produces the corresponding point reputation as output.

**Figure 2:**
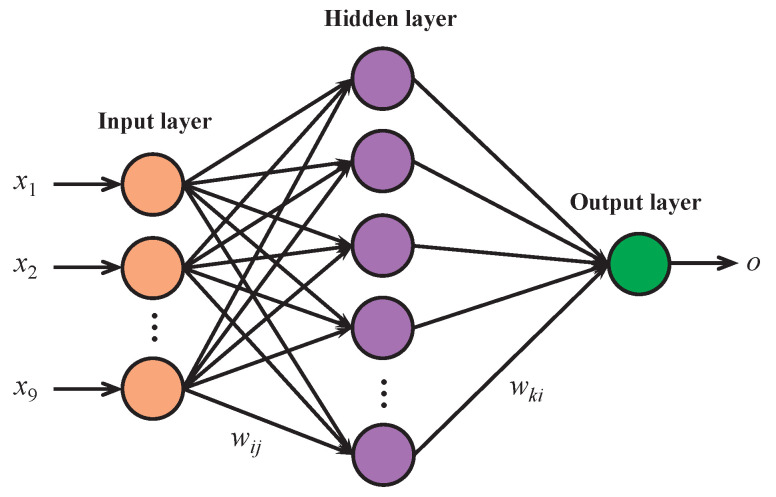
Diagram of our designed BPNN model.

The BPNN model we designed for point reputation evaluation is shown in Figure 2, which consists of an input layer, a hidden layer, and an output layer. Specifically, we represent the input vector of the input layer as $x=\left(x_{1},x_{2},\ldots ,x_{9}\right)$, where the nine elements correspond to the nine evaluation indexes defined in Table 1. Accordingly, the input layer consists of nine neurons. Because our BPNN model outputs only one value for the point reputation evaluation result, the output layer has only one neuron. We denote the input of the output layer by *z*, and the actual output result and the expected output result are denoted by *o* and *g*, respectively. The number of neurons in the hidden layer is denoted by *l*, and the input and output vectors of the hidden layer are denoted by $f=\left(f_{1},f_{2},\ldots ,f_{l}\right)$ and $y=\left(y_{1},y_{2},\ldots ,y_{l}\right)$, respectively. It is important to choose an appropriate number of neurons in the hidden layer to optimize the accuracy and efficiency of the BPNN model. Most existing methods determine the number of neurons in the hidden layer by trial and error. Typically, an approximate range of neuron numbers is first set based on prior experience. Then, each number within this range is tested in the BPNN model while keeping all other conditions constant, and the best result is used to determine the required number of neurons in the hidden layer. The number of neurons in the hidden layer is generally related to the number of neurons in the input and output layers. The formulas commonly used to determine the approximate range of neuron numbers in the hidden layer are as follows:

(1) $$l=\sqrt{p+q}+\lambda ,$$

(2) $$l=\sqrt{pq},$$

(3) $$l=\log_{2}p,$$

where *p* and *q* are the number of neurons in the input and output layers, respectively, and λ is a constant within $\left[1,10\right]$. Applying these formulas to our BPNN model with p=9 and q=1, we can calculate the range of hidden layer neuron numbers for our BPNN model as $\left[3,14\right]$. The optimal choice of the number of neurons in the hidden layer still needs to be determined by network training with dynamic debugging.

Next, we present the operation process of the BPNN model to demonstrate the sensitivity and effectiveness of the heuristic used in translating evaluation indexes into the point reputation.

(1) Initialization: We denote the connection weight from input layer neuron *j* to hidden layer neuron *i* as $w_{ij}$. The threshold at hidden layer neuron *i* is denoted by $a_{i}$, and the activation function at the hidden layer is denoted by $\varphi \left(\cdot \right)$. Moreover, we denote the connection weight from hidden layer neuron *i* to output layer neuron *o* as $w_{ko}$. The threshold at output layer neuron *o* is denoted by $b_{o}$, and the activation function at the output layer is denoted by $\psi \left(\cdot \right)$. In the initialization process, $w_{ij}$ and $w_{ko}$ are assigned random values, $a_{i}$ and $b_{o}$ are set to 0, and a learning rate η is specified. The total number of training iterations is also set to a fixed value to ensure the network can terminate training.

(2) Forward Propagation: The input layer forwards the external information it receives to the hidden layer. The input expression at hidden layer neuron *i* is as follows:

(4) $$f_{i}=\sum_{j=1}^{9}w_{ij}x_{j}-a_{i}.$$

The output of the hidden layer neuron *i* is then obtained by applying the activation function $\varphi \left(\cdot \right)$ to the input $f_{i}$:

(5) $$y_{i}=\varphi \left(f_{i}\right).$$

The input expression at the output layer neuron *o* is obtained by summing the products of the hidden layer neuron outputs $y_{i}$ and their corresponding weights $w_{ko}$:

(6) $$z=\sum_{i=1}^{l}w_{ko}y_{i}-b_{o}.$$

Finally, the output of the output layer neuron *o* is obtained by applying the activation function $\psi \left(\cdot \right)$ to the input *z*:

(7) $$o=\psi \left(z\right).$$

(3) Backpropagation: Comparing the output obtained from the output layer with the expected output determines the error. If the error is not within the expected range or the training is ongoing, the adjustment of the weights and thresholds between the output layer and the hidden layer, as well as between the hidden layer and the input layer, is made by propagating the error backward from the output layer to the input layer. The training continues for multiple iterations until the output result is within the expected range or the maximum number of iterations is reached. The error function is expressed by Equation (8), where *K* represents the number of samples.

(8) $${\mathrm{Error}}_{\mathrm{BP}}=\frac{1}{2K}\sum_{k=1}^{K}{\left(o_{k}-g_{k}\right)}^{2}.$$

### 4.3. Credibility and Global Reputation Calculation

To detect malicious devices that launch single-point attacks and ensure the accuracy of global reputations, we construct a visualization model PR-WDNM for the reputation evaluation network. We then calculate credibility and global reputation based on our PR-WDNM.

#### 4.3.1. PR-WDNM

Considering the relationships between devices in the point reputation evaluation, we construct the PR-WDNM, which assigns weights to the edges in the reputation network with determined directions.

**Figure 3:**
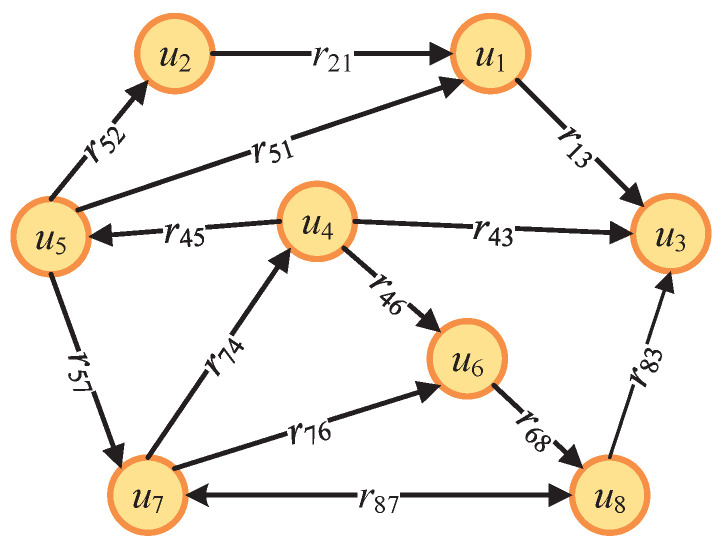
Diagram of our designed PR-WDNM.

As shown in Figure 3, our PR-WDNM can be denoted by a triple $\left(U,E,R\right)$, where $U=\{u_{1},u_{2},\cdots ,u_{n}\}$ denotes the set of all intelligent inspection devices in an IoT-enabled PSPS, $E=\{e_{1},e_{2},\cdots ,e_{m}\}$ denotes the set of point reputation evaluation relationships, and *R* denotes the weight matrix of directed edges. Moreover, $\left(u_{i},u_{j}\right)\in E$ denotes a weighted directed edge from $u_{i}$ to $u_{j}$, and $r_{ij}$ denotes the weight of $\left(u_{i},u_{j}\right)$, which is equal to the point reputation value evaluated by $u_{i}$ to $u_{j}$. By performing this, we visually illustrate the point reputation evaluation relationships between devices. Moreover, the statistical properties in PR-WDNM include the evaluation relationships between devices, point reputation values of each device, average point reputation values of all devices, as well as the sets of evaluator devices and evaluatee devices. They will be utilized in the credibility and global reputation calculations to detect malicious devices that launch single-point attacks.

#### 4.3.2. Credibility Calculation

To calculate the credibility $c_{i}$ of $u_{i}$, we first calculate the average point reputation of each device. By analyzing the statistical properties of our PR-WDNM, the average point reputation ${\bar{r}}_{j}$ of $u_{j}$ is equivalent to the average value of the weights on all edges pointing to $u_{j}$, which can be expressed as

(9) $${\bar{r}}_{j}=\frac{\sum_{u_{k}\in U_{j}^{\mathrm{rec}}}r_{kj}}{\left|U_{j}^{\mathrm{rec}}\right|},$$

where $U_{j}^{\mathrm{rec}}$ denotes the set of devices that provide point reputation evaluation to $u_{j}$, which is equivalent to the set of all nodes pointing to $u_{j}$ in our PR-WDNM. $u_{k}$ denotes the *k*-th device in $U_{j}^{\mathrm{rec}}$, k=1,2,3,…, and $r_{kj}$ denotes the point reputation of $u_{j}$ evaluated by $u_{k}$. Next, we define the credibility $c_{i}$ of $u_{i}$ as

(10) $$c_{i}=\frac{\sum_{u_{j}\in U_{i}^{\mathrm{sen}}}\frac{r_{ij}}{{\bar{r}}_{j}}}{\left|U_{i}^{\mathrm{sen}}\right|},$$

where $U_{i}^{\mathrm{sen}}$ denotes the set of devices evaluated by $u_{i}$, which is equivalent to the set of nodes pointed by $u_{i}$ in our PR-WDNM. $u_{j}$ denotes the *j*-th device in $U_{i}^{\mathrm{sen}}$, j=1,2,3,…, and $r_{ij}$ denotes the point reputation of $u_{j}$ evaluated by $u_{i}$.

Furthermore, we utilize $c_{i}$ to detect malicious devices that post false point reputations. The average value of all devices’ credibilities can be calculated as

(11) $$\mu =\frac{\sum_{i=1}^{n}c_{i}}{n},$$

where *n* denotes the number of devices and μ can reflect the credibility level of the whole network, which is the basis for malicious device judgment. Specifically, if $c_{i}$ is less than μ, $u_{i}$ will be judged as a malicious device that has posted false point reputations.

#### 4.3.3. Global Reputation Calculation

Each device’s global reputation is obtained by aggregating its point reputations evaluated by others. Before aggregation, we remove the false point reputations evaluated by the detected malicious devices. Next, we utilize the devices’ normalized credibilities as the weights to calculate the average of the point reputation values as the global reputation of the device. This weighted average method effectively mitigates the impact of abnormal point reputations provided by low-credibility devices on the global reputation calculation. When calculating the global reputation $R_{k}$ of $u_{k}$, the process of normalizing $c_{i}$ of $u_{i}$ into its weight $\beta_{i}$ can be expressed as

(12) $$\beta_{i}=\frac{c_{i}}{\sum_{u_{j}\in U_{k}^{\mathrm{rec}}}c_{j}},$$

where $U_{k}^{\mathrm{rec}}$ denotes the set of devices that provide point reputation evaluation to $u_{k}$ and $u_{i}\in U_{k}^{\mathrm{rec}}$. Therefore, $R_{k}$ can be calculated as

(13) $$R_{k}=\frac{\sum_{u_{i}\in U_{k}^{\mathrm{rec}}}\beta_{i}\cdot r_{ik}}{\left|U_{k}^{\mathrm{rec}}\right|}.$$

Based on Equation (13), devices with low global reputations can be identified as malicious, indicating that they provide bad services. Additionally, global reputations can serve as reliable references for future interactions, thereby ensuring a secure and trustworthy PSPS IoT environment.

## 5. Collusion Device Identification Methods

In this section, we propose a knowledge graph-based collusion device identification method. By constructing a knowledge graph, we can measure the degree of associations between devices and calculate their behavioral and semantic similarities using vectorized knowledge. This allows us to identify collusion devices with the same malicious behaviors.

### 5.1. Knowledge Graph Design

The knowledge graph design comprises two significant components: entity attribute selection and knowledge vectorization. According to the common characteristics of collusion devices, we first select multiple attributes of the devices for constructing the knowledge graph. Subsequently, we introduce the knowledge vectorization method.

#### 5.1.1. Entity Attributes

Collusion devices involved in collusion attacks often share common traits and engage in coordinated actions to manipulate the reputation evaluation system in IoT-enabled PSPSs. Based on the threat model in Section 3.2, we present the following assumptions for the collusion attacks. First, in PSPS scenarios where large numbers of intelligent inspection devices are deployed, a collusion attack requires multiple devices to be involved in the collusion to ensure the attack is effective and damaging. Second, to maintain a facade of legitimacy, collusion devices work together to present a unified front. They maintain consensus in trusted objects, untrusted objects, and potential attack targets. Third, collusion devices tend to employ similar attack methods, such as slandering the reputations of others or exaggerating the reputations of their partners, often with a similar number of evaluations. Through these coordinated actions, colluding devices generate similar global reputations and credibility. Fourth, collusion devices maintain close communication with constant objects to ensure the successful execution of the attack. Therefore, by collaborating closely, collusion devices can coordinate their efforts and align their behaviors to deceive the reputation evaluation system. Based on the characteristics of collusion attacks and the behaviors exhibited by colluding devices, we select several device attributes to construct the knowledge graph, as shown in Table 2.

We adopt the Protégé tool to build the ontology and Neo4j to store the knowledge graph. In Neo4j, nodes represent entities in the knowledge graph, and edges represent relationships between entities. The configurations of our knowledge graph in Neo4j are shown in Table 3.

#### 5.1.2. Knowledge Vectorization

We apply the TransE algorithm to map entities and their relationships in the knowledge graph to a low-dimensional vector space, aiming to facilitate lightweight similarity calculation. Specifically, we construct a triple $\left(v_{h},r,v_{t}\right)$, where $v_{h}$ denotes the head entity, $v_{t}$ denotes the tail entity, and *r* denotes the relationship between the two entities. This triple is embedded in a *d*-dimensional vector space $R^{d}$, where $v_{h},v_{t},r\in R^{d}$. The TransE algorithm connects $v_{h}$ and $v_{t}$ in the knowledge graph through *r*, and this process follows $v_{h}+r\approx v_{t}$. In fact, there may be embedding errors when representing entities and relations as vectors during the embedding process in a knowledge graph. To quantify this error, we utilize the L2 norm to calculate the difference between the head entity vector, tail entity vector, and the relation vector during the conversion process. We define the embedding error function for a single triple as

(14) $$F\left(v_{h},r,v_{t}\right)={∥v_{h}+r-v_{t}∥}_{2}^{2},$$

where ${\left|\cdot \right|}_{2}^{2}$ denotes the square of the L2 norm of a vector. This embedding error indicates the degree of difference between the head entity vector, relation vector, and tail entity vector, i.e., the Euclidean distance between them in the vector space. A smaller embedding error suggests that the vectors are closer in distance, indicating higher embedding quality for the triple. Conversely, a larger embedding error suggests a greater distance between the vectors, indicating lower embedding quality for the triple. Next, we define the objective function for all triples in the knowledge graph based on Equation (14), which can be expressed as

(15) $$L=\sum_{\chi \in I}\sum_{\chi^{\prime }\in I^{\prime }}\max\left(0,F\left(v_{h},r,v_{t}\right)-F\left(v_{h^{\prime }},r,v_{t^{\prime }}\right)+\gamma \right),$$

where $\chi =\left(v_{h},r,v_{t}\right)$ and $\chi^{\prime }=\left(v_{h^{\prime }},r,v_{t^{\prime }}\right)$, respectively, denote the correct and incorrect triples in our knowledge graph; *I* and $I^{\prime }$, respectively, denote the correct and incorrect triple sets; and γ denotes the distance parameter between *I* and $I^{\prime }$. The correct triple refers to a triple that exists in the knowledge graph, where the head entity, tail entity, and relation type are all correct. On the other hand, the incorrect triple refers to a triplet that does not exist in the knowledge graph, where at least one of the head entity, tail entity, or relation type is incorrect. Incorrect triples are used as negative samples for training knowledge graph models. Our TransE algorithm is trained by Equation (15), aiming to minimize the distance gap between the correct and incorrect triples, thereby improving the quality of the embedding. By doing so, we can obtain a vectorized description of the entities and relationships in the knowledge graph to support lightweight similarity computation.

### 5.2. Similarity Calculation

To identify collusion behaviors of malicious devices, it is important to quantify the similarity between devices and identify those with similar attack behaviors. Therefore, we compute the behavioral similarity based on devices’ point reputations and semantic similarity based on the vector relationships in our knowledge graph. The fusion of both similarities is used for collusion device identification.

#### 5.2.1. Behavioral Similarity Calculation

The devices in a collusion group have the same attack targets and behaviors, resulting in similar point reputations of the targets evaluated by the collusion devices. In this regard, we define the similarity of point reputation evaluation behaviors between devices as the behavioral similarity of devices. The point reputation matrix of the mutual evaluation between devices can be expressed by

(16) $$R_{n\times n}=\left[\begin{array}{cccc} r_{11} & r_{12} & \cdots & r_{1n}\\ r_{21} & r_{22} & \cdots & r_{2n}\\ \cdots & \cdots & \cdots & \cdots \\ r_{n1} & r_{n2} & \cdots & r_{nn} \end{array}\right],$$

where the matrix element $r_{ij}$ denotes the point reputation of $u_{j}$ evaluated by $u_{i}$. According to Equation (16), the point reputation evaluation vectors of $u_{i}$ and $u_{j}$ can be expressed as $S_{i}=\left(r_{i1},r_{i2},\cdots ,r_{in}\right)$ and $S_{j}=\left(r_{j1},r_{j2},\cdots ,r_{jn}\right)$, respectively. The factors in the vector denote the point reputations evaluated by a device for others. We use the cosine similarity between $S_{i}$ and $S_{j}$ as the behavioral similarity ${\mathrm{bs}}_{ij}$ between $u_{i}$ and $u_{j}$, which can be expressed as

(17) $${\mathrm{bs}}_{ij}=\frac{S_{i}\cdot S_{j}}{∥S_{i}∥\cdot ∥S_{j}∥}=\frac{\sum_{k=1}^{n}r_{ik}\cdot r_{jk}}{\sqrt{\sum_{k=1}^{n}r_{ik}^{2}}\cdot \sqrt{\sum_{k=1}^{n}r_{jk}^{2}}}.$$

Furthermore, the behavioral similarity matrix can be expressed as

(18) $${\mathrm{BS}}_{n\times n}=\left[\begin{array}{cccc} {\mathrm{bs}}_{11} & {\mathrm{bs}}_{12} & \cdots & {\mathrm{bs}}_{1n}\\ {\mathrm{bs}}_{21} & {\mathrm{bs}}_{22} & \cdots & {\mathrm{bs}}_{2n}\\ \cdots & \cdots & \cdots & \cdots \\ {\mathrm{bs}}_{n1} & {\mathrm{bs}}_{n2} & \cdots & {\mathrm{bs}}_{nn} \end{array}\right].$$

#### 5.2.2. Semantic Similarity Calculation

Based on our constructed knowledge graph, $u_{i}$ can be represented by a *d*-dimensional vector as

(19) $$u_{i}={\left(\pi_{1i},\pi_{2i},\cdots ,\pi_{di}\right)}^{T},$$

where $\pi_{ki}$ denotes the value of the vector embedded by $u_{i}$ in the *k*-th dimension, k=1,2,…,d. Next, we normalize the L2 norm of $u_{i}$ and $u_{j}$ to obtain the knowledge graph-based semantic similarity ${\mathrm{gs}}_{ij}$ between $u_{i}$ and $u_{j}$, which can be expressed as

(20) $${\mathrm{gs}}_{ij}=\frac{1}{1+{∥u_{i}-u_{j}∥}_{2}}=\frac{1}{1+\sqrt{\sum_{k=1}^{d}{\left(\pi_{ki}-\pi_{kj}\right)}^{2}}}.$$

Furthermore, the semantic similarity matrix can be expressed as

(21) $${\mathrm{GS}}_{n\times n}=\left[\begin{array}{cccc} {\mathrm{gs}}_{11} & {\mathrm{gs}}_{12} & \cdots & {\mathrm{gs}}_{1n}\\ {\mathrm{gs}}_{21} & {\mathrm{gs}}_{22} & \cdots & {\mathrm{gs}}_{2n}\\ \cdots & \cdots & \cdots & \cdots \\ {\mathrm{gs}}_{n1} & {\mathrm{gs}}_{n2} & \cdots & {\mathrm{gs}}_{nn} \end{array}\right].$$

#### 5.2.3. Similarity Fusion

We obtain behavioral similarity based on the point reputation evaluation results and the semantic similarity based on the vectors of entities and their relations in the knowledge graph. To identify malicious devices with collusive behaviors, we fuse the two similarity measures to obtain a comprehensive similarity that takes into account device attributes, relationships, and reputation evaluation behaviors. The fusion process can be expressed as

(22) $${\mathrm{fs}}_{ij}=\rho \cdot {\mathrm{gs}}_{ij}+\left(1-\rho \right)\cdot {\mathrm{bs}}_{ij},$$

where ${\mathrm{fs}}_{ij}$ denotes the fused similarity between $u_{i}$ and $u_{j}$ and $\rho \in \left[0,1\right]$ is the fusion weight. As such, the fused similarity matrix can be further expressed as

(23) $${\mathrm{FS}}_{n\times n}=\left[\begin{array}{cccc} {\mathrm{fs}}_{11} & {\mathrm{fs}}_{12} & \cdots & {\mathrm{fs}}_{1n}\\ {\mathrm{fs}}_{21} & {\mathrm{fs}}_{22} & \cdots & {\mathrm{fs}}_{2n}\\ \cdots & \cdots & \cdots & \cdots \\ {\mathrm{fs}}_{n1} & {\mathrm{fs}}_{n2} & \cdots & {\mathrm{fs}}_{nn} \end{array}\right].$$

To detect collusion devices, we set two special judgment thresholds. Specifically, we first introduce a threshold ε for the fused similarity. If ${\mathrm{fs}}_{ij}$ is greater than ε, then $u_{i}$ and $u_{j}$ are identified as suspicious collusion devices. We further introduce a threshold δ for the total number of collusion devices. When the total number of devices with similar fused similarity is greater than δ, these devices are identified as collusion devices.

## 6. Simulation Results

In this section, we validate the performance of our *ReIPS* for the reputation evaluations of intelligent inspection devices by extensive simulations. Our *ReIPS* is implemented on a Windows 10 system with a Python 3.6 environment. We provide the simulation settings and results in detail below.

### 6.1. Simulation Settings

During simulations, we use the parameter settings summarized in Table 4 to ensure the stability, convergence, and precision of our *ReIPS*. The BPNN model comprises three layers, with sigmoid and ReLU activation functions used in the hidden and output layers, respectively. We use the quadratic loss function as the model’s loss function.

To compare the performance of our *ReIPS* in single-point attack scenarios, we use two existing methods as benchmarks in the simulation: the reputation measurement method (RM) in [30] and the trust evaluation method (TE) in [31]. RM calculates the global reputation of a device by averaging all point reputations evaluated by others, whereas TE_1 and TE_2 correct the global reputation by assigning low weights to extreme evaluations and high weights to normal evaluations, respectively. For comparison in collusion attack scenarios, we consider two benchmarks: the reputation evaluation method (BS-RE), which only considers behavioral similarity in identifying collusion devices [32], and our *ReIPS* without the proposed collusion device identification method (ReIPS-NoCDI).

We use four metrics to evaluate the performance of reputation evaluation: mean absolute error (MAE) and mean square error (MSE) for global reputation evaluation results, and precision and recall for global reputation evaluation methods, which are expressed as

(24) $$\mathrm{MAE}=\frac{\sum_{i=1}^{n}\left|R_{i}-{\hat{R}}_{i}\right|}{n},$$

(25) $$\mathrm{MSE}=\frac{\sum_{i=1}^{n}{\left(R_{i}-{\hat{R}}_{i}\right)}^{2}}{n},$$

(26) $$\mathrm{Precision}=\frac{TP}{TP+FP},$$

(27) $$\mathrm{Recall}=\frac{TP}{TP+FN},$$

where *n* is the number of devices, $R_{i}$ is the true global reputation value of $u_{i}$, and ${\hat{R}}_{i}$ is the global reputation value evaluated by each method. The definitions of TP, FP, and FN are shown in Table 5. Normal devices are programmed to provide point reputations with an error of no more than 10%, whereas abnormal devices are programmed to provide point reputations with an error of over 10%. Abnormal devices that participate collusion attacks are further configured to have the same trust object set, distrust object set, and communication object set to exhibit their colluding behaviors. In addition, we consider a range of [20, 100] for the total number of intelligent inspection devices in a PSPS, where the proportion of malicious devices ranges from 5% to 45%.

### 6.2. Performance in Single-Point Attack Scenarios

#### 6.2.1. Performance with Different Percentages of Malicious Devices

**Figure 4:**
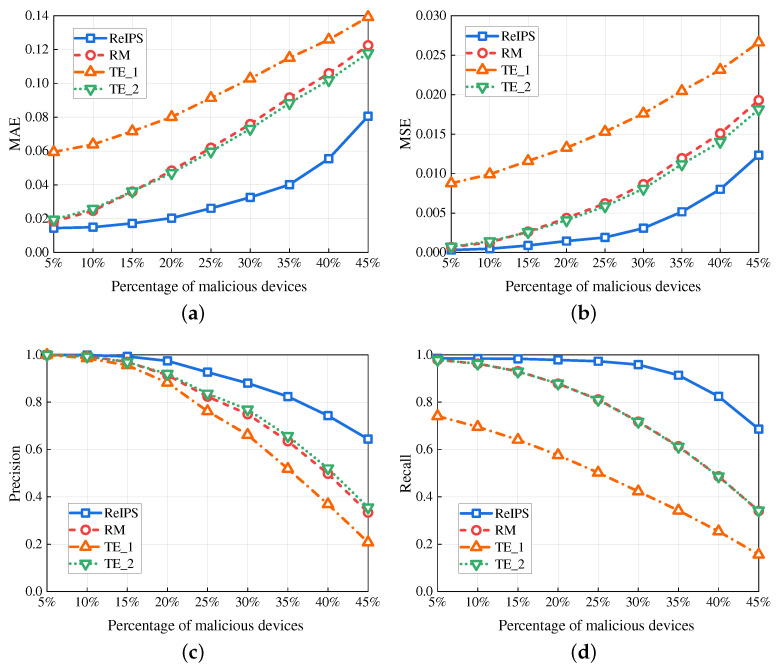
Performance in different methods versus the percentage of malicious devices. (**a**) MAE. (**b**) MSE. (**c**) Precision. (**d**) Recall.

Figure 4 plots the four performance metrics for different methods versus the percentage of malicious devices, with a fixed total number of 100 devices. As depicted in Figure 4a,b, our *ReIPS* achieves lower MAE and MSE compared to the benchmarks. For example, when the percentage of malicious devices is 30%, the MAE of our *ReIPS* is 42.96% for RM, 30.7% for TE_1, and 44.62% for TE_2, whereas the MSE of our *ReIPS* is 35.57% for RM, 17.43% for TE_1, and 38.09% for TE_2 This can be attributed to our *ReIPS* calculating the device credibility based on point reputations and using it as the weight of point reputation for global reputation aggregation. Specifically, the point reputation evaluated by a normal device carries a larger weight, whereas the point reputation evaluated by an abnormal node carries a smaller weight or even 0. Consequently, our *ReIPS* improves the reliability of the global reputation evaluation results. Figure 4c,d demonstrate that our *ReIPS* outperforms the benchmarks in precision and recall. When the percentage of malicious devices is 30%, the precision of our *ReIPS* is 0.13, 0.12, and 0.11 higher than that of RM, TE_1, and TE_2, respectively, and the recall of our *ReIPS* is 0.24, 0.54 and 0.24 higher than that of RM, TE_1, and TE_2, respectively. This is because our proposed PR-WDNM can identify the malicious devices launching single-point attacks and filter out their false point reputations, thereby removing their impact on the global reputation calculation.

#### 6.2.2. Performance with Different Total Numbers of Devices

**Figure 5:**
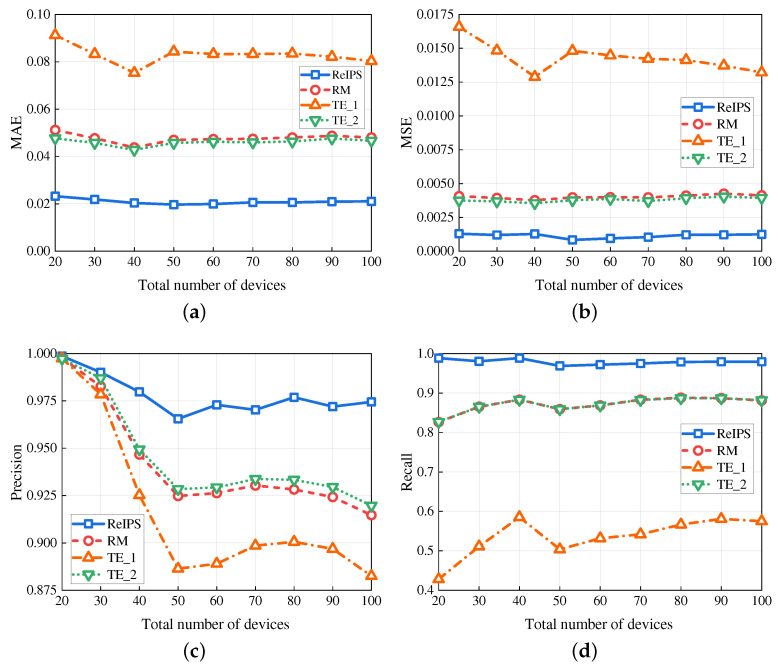
Performance in different methods versus the total number of devices. (**a**) MAE. (**b**) MSE. (**c**) Precision. (**d**) Recall.

Figure 5 shows the four performance metrics for different methods versus the total number of devices with a fixed percentage of 20% for malicious devices. Figure 5a,b demonstrate that our *ReIPS* has consistently lower MAE and MSE compared to the benchmark algorithms. For example, when the total number of devices is 60, the MAE of our *ReIPS* is 42.13% for RM, 23.95% for TE_1, and 43.17% for TE_2, whereas the MSE of our *ReIPS* is 23.75% for RM, 6.55% for TE_1, and 24.55% for TE_2. Moreover, both figures also shows that the MAE and MSE hardly change as the number of devices increases. This is because our *ReIPS* ensures the correctness of global reputation aggregation by calculating device credibility based on point reputations, which is unaffected by the total number of devices. Figure 5c,d exhibit the superior performance of our *ReIPS* in terms of precision and recall. When the total number of devices is 60, the precision of our *ReIPS* is 0.05, 0.08, and 0.04 higher than the values of RM, TE_1, and TE_2, respectively, and the recall of our *ReIPS* is 0.1, 0.44 and 0.1 higher than the values of RM, TE_1, and TE_2, respectively. This is because our *ReIPS* can accurately detect and filter false point reputations generated by malicious devices. Furthermore, Figure 5 demonstrates that our *ReIPS* maintains good performance stability as the number of devices increases, making it suitable for IoT-enabled PSPSs with a large number of intelligent inspection devices.

### 6.3. Performance in Collusion Attack Scenarios

**Figure 6:**
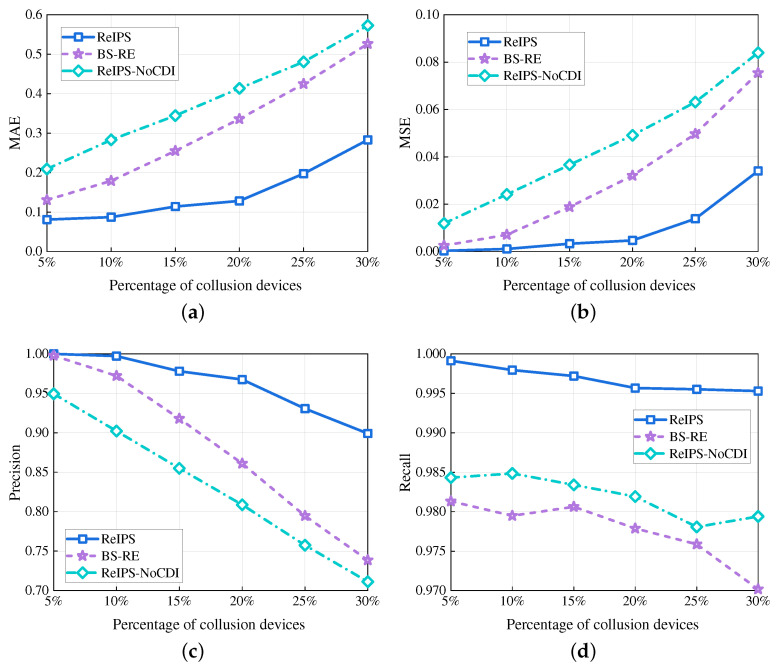
Performance in different methods versus the percentage of collusion devices. (**a**) MAE. (**b**) MSE. (**c**) Precision. (**d**) Recall.

Figure 6 plots the four performance metrics for different methods versus the percentage of collusion devices, with a fixed total of 100 devices. As shown in Figure 6a–d, our *ReIPS* consistently outperforms the benchmarks with lower MAE and MSE, and has a higher precision and recall as the percentage of collusion devices increases. For example, when the percentage of collusion devices is 20%, the MAE and MSE of our *ReIPS* are 38.16% and 14.66% for BS-RE, and 31% and 9.57% for ReIPS-NoCDI, respectively. Regarding precision and recall, our *ReIPS* obtained values 0.11 and 0.018 higher than BS-RE, and 0.16 and 0.014 higher than ReIPS-NoCDI, respectively. Unlike ReIPS-NoCDI, our *ReIPS* considers the impact of malicious evaluation behaviors from collusion devices on global reputation aggregation. Our proposed knowledge graph-based collusion device identification method accurately detects collusion devices, filters out false point reputations, and removes devices providing bad services from the IoT network. This ensures the correctness of global reputation evaluation under collusion attack scenarios. Moreover, compared to BS-RE, our *ReIPS* considers both behavioral and semantic similarities between devices, enhancing its collusion device identification ability. Therefore, our *ReIPS* can ensure secure reputation evaluation in IoT-enabled PSPSs with single-point and collusion attack scenarios.

## 7. Conclusions

This paper presented *ReIPS*, a secure cloud-based reputation evaluation system designed to maintain a trusted IoT environment comprising intelligent inspection devices in PSPSs. We first introduced a cloud platform for IoT-enabled PSPSs to handle resource-consuming operations related to reputation evaluation. Then we proposed a novel reputation evaluation model based on BPNN and PR-WDNM to improve the precision and objectivity of reputation evaluation. Our PR-WDNM was used to detect malicious devices that launch single-point attacks and improve the correctness of global reputation aggregation. Moreover, we proposed a knowledge graph-based collusion device identification method that utilizes both behavioral and semantic similarities to accurately detect colluding devices and prevent their impact on global reputation aggregation. Simulation results demonstrate that our *ReIPS* exhibits good performance and usability in IoT-enabled PSPSs. Future work is in progress to apply our ReIPS in real PSPSs and evaluate its universality with more experimental parameter adjustments related to the network status, multi-type attacks, and improved learning algorithms. 

## Figures and Tables

**Table 1:** Definitions of reputation evaluation indexes.

Index	Definition
Specification	The degree to which the service description language document conforms to the specification of the service description language.
Agreement	The degree to which the service follows the network service agreement profile.
Description	The metric for service description language documentation.
Response time	The time span between when a device makes an interaction request and when it receives a response.
Throughput	The maximum number of requests processed in a given unit of time.
Latency	The time required to process the given request.
Link success rate	The ratio of the number of response messages to the number of request messages.
Availability	The ratio of the number of successful calls to the total number of calls.
Reliability	The ratio of the number of correct messages to the total number of messages.

**Table 2:** Attributes of knowledge graph.

Attribute	Type
Device Number	data
Global reputation	data
Credibility	data
Number of evaluations	data
Trusted objects	object
Untrusted objects	object
Communication objects	object

**Table 3:** Configurations in Neo4j.

Item	Content
Node	Device entity
Edge	Trusted, untrusted, and communication relationships
Node attribute	Device number, global reputation, credibility, and number of evaluations

**Table 4:** Parameter settings.

Parameter	Value
BPNN iterations	20,000
BPNN learning rate	0.1
Number of input layer nodes	9
Number of output layer nodes	1
Number of hidden layer nodes	13
Embedding dimension, *d*	150
Similarity fusion weight, ρ	0.7

**Table 5:** Confusion matrix.

	Actual Normal Devices	Actual Abnormal Devices
Identified normal devices	TP	FP
Identified abnormal devices	FN	TN

## Data Availability

Data sharing not applicable.
